# Profiles of overall survival-related gene expression-based risk signature and their prognostic implications in clear cell renal cell carcinoma

**DOI:** 10.1042/BSR20200492

**Published:** 2020-09-16

**Authors:** Zihao He, Tuo Deng, Xiaolu Duan, Guohua Zeng

**Affiliations:** 1 Department of Urology and Guangdong Key Laboratory of Urology, The First Affiliated Hospital of Guangzhou Medical University, Guangzhou, Guangdong, China, 510230; 2 Guangzhou Institute of Urology, Guangzhou, China

**Keywords:** bioinformatic analysis, clear cell renal carcinoma, prognosis

## Abstract

The present work aimed to evaluate the prognostic value of overall survival (OS)-related genes in clear cell renal cell carcinoma (ccRCC) and to develop a nomogram for clinical use. Transcriptome data from The Cancer Genome Atlas (TCGA) were collected to screen differentially expressed genes (DEGs) between ccRCC patients with OS > 5 years (149 patients) and those with <1 year (52 patients). In TCGA training set (265 patients), seven DEGs (cytochrome P450 family 3 subfamily A member 7 (CYP3A7), contactin-associated protein family member 5 (CNTNAP5), adenylate cyclase 2 (ADCY2), TOX high mobility group box family member 3 (TOX3), plasminogen (PLG), enamelin (ENAM), and collagen type VII α 1 chain (COL7A1)) were further selected to build a prognostic risk signature by the least absolute shrinkage and selection operator (LASSO) Cox regression model. Survival analysis confirmed that the OS in the high-risk group was dramatically shorter than their low-risk counterparts. Next, univariate and multivariate Cox regression revealed the seven genes-based risk score, age, and Tumor, lymph Node, and Metastasis staging system (TNM) stage were independent prognostic factors to OS, based on which a novel nomogram was constructed and validated in both TCGA validation set (265 patients) and the International Cancer Genome Consortium cohort (ICGC, 84 patients). A decent predictive performance of the nomogram was observed, the C-indices and corresponding 95% confidence intervals of TCGA training set, validation set, and ICGC cohort were 0.78 (0.74–0.82), 0.75 (0.70–0.80), and 0.70 (0.60–0.80), respectively. Moreover, the calibration plots of 3- and 5 years survival probability indicated favorable curve-fitting performance in the above three groups. In conclusion, the proposed seven genes signature-based nomogram is a promising and robust tool for predicting the OS of ccRCC, which may help tailor individualized therapeutic strategies.

## Introduction

As one of the most common urinary malignancies, renal cell carcinoma (RCC) poses a hidden threat to public health and accounts for approximately 2–3% of adult tumors [1]. The main histologic subtype of RCC is clear cell RCC (ccRCC), which constitutes 75–80% of primary renal malignancies [2]. Reportedly, ccRCC generated 65340 newly diagnosed cases and 14970 deaths in America in 2018 [3].

Compared with other tumors, the prognosis of ccRCC patients remains generally preferable as indicated by the 5-year overall survival (OS) of localized (stage I–III) ccRCC had reached up to 70–90% [4]. Despite this, individual variations should be recognized as patients with similar Tumor, lymph Node, and Metastasis staging system (TNM) stages at diagnosis could end up with significantly different OS. For example, 25–33% of localized ccRCC patients could still progress into recurrence and metastasis even after curative resection and associated with a significantly worse prognosis than other patients with localized ccRCC [5,6]. Besides, the morphologic and genetic heterogeneity of ccRCC was discussed in numerous previous studies [7,8]. Such reports suggested that the prevalent prognostic tools for ccRCC, which were based mainly on pathological and clinical features, had unsatisfactory predictive power. Typical tools of this kind included the TNM staging system, necrosis score, and the University of California Integrated Staging System (UISS) [9–11].

Today, with the development of high-throughput sequencing technology, urologists have turned to identify molecular biomarkers for risk stratification and prognosis prediction. In this regard, prognostic tools from a single gene [12–14], to risk signatures consist of a panel of genes [15–17], and to models integrating gene profiling and clinical features [18–20], have been widely reported. However, there has been no study yet in this field to investigate genes correlated directly to ccRCC patients’ OS and to explore their prognostic values in medical practice, and we believe that data mining in this topic could provide new insight into ccRCC progression and help improve therapeutic strategies to a great extent.

## Materials and methods

### Data acquisition

The transcriptome profiling data and clinical information of ccRCC were obtained from The Cancer Genome Atlas (TCGA, https://portal.gdc.cancer.gov/) in December 2019. Expression data as Fragments Per Kilobase per Million (FPKM) files were available for 539 tumor samples, 9 of which lacked survival information. The 530 samples with full expression and clinical data were randomly and evenly assigned to a training set and a validation set via the ‘Classification and Regression Training (caret)’ package (http://topepo.github.io/caret/) in R (Ver. 3.6.0) for further analysis. Besides, 84 ccRCC patients with full data from the International Cancer Genome Consortium (ICGC, https://icgc.org/) were used as external validation. The OS (or time to death), defined as the time from the start of follow-up (surgery) to death of any cause, was regarded as the target event.

### Screening for survival-related genes

In TCGA, 52 patients with OS < 1 year and 149 patients with OS > 5 years were enrolled in the differential expression analysis via the limma package in R to screen out survival-related differentially expressed genes (DEGs) and acquire corresponding fold changes (FCs). Specifically, DEGs with |log_2_FC| > 0.60 and false discovery rate (FDR) < 0.05 were considered to be the hub DEGs. Outcomes were visualized as volcano plot and heatmap using ‘ggplot2’ and ‘pheatmap’ packages in R, respectively.

### Functional enrichment analysis

After ruling out the non-coding RNAs, the rest DEGs with protein-coding functions were analyzed by the Database for Annotation Visualization and Integrated Discovery (DAVID; https://david.ncifcrf.gov/) online tools to obtain their Gene Oncology (GO) and Kyoto Encyclopedia of Genes and Genomes (KEGG) pathway enrichment information. The criteria were set as *P*<0.05 and gene count ≥3.

### Prognostic risk signature development

The hub DEGs with protein-coding functions were selected as candidates for the analysis of this section. In the training set, the univariable Cox regression analysis using ‘survival’ package in R was primarily performed to filter insignificant candidates (*P*>0.05). Next, the least absolute shrinkage and selection operator (LASSO) Cox regression method [21] using ‘glmnet’ and ‘survival’ packages was performed to select the optimal panel of genes included in the risk score formula. Last, the multivariate Cox regression analysis was used to obtain the coefficients of each included gene. The risk score of each patient was equal to the sum of the products of each gene’s expression value (Exp_i_) and the corresponding coefficients. Using the median score as the cut-off point, ccRCC patients were divided into a low-risk group and high-risk group. The Kaplan–Meier survival analysis with a log-rank test, and the area under the curve (AUC) of the receiver operating characteristic (ROC) curve were used as an initial evaluation of the risk signature.

### Construction and validation of nomogram for OS prediction

To identify independent prognostic factors to OS, parameters including age, gender, TNM stage, history of prior malignancy, and the aforesaid risk score were included in univariate and multivariate Cox regression analyses in the training set. Using the ‘rms’ package in R, a nomogram incorporating all the significant factors (*P*<0.05) was constructed to predict the 3- and 5-years OS. For predictive performance assessment, Harrell’s concordance index (C-index) and calibration plot were obtained in training and validation sets. Similar to the AUC of ROC curve, C-index using a bootstrap method with 1000 resamplings was calculated to assess the discriminatory ability of nomogram [22,23]. The calibration plot compared the observed and predicted probabilities, and the 45-degree line represents the highest predictive ability.

## Results

### Patient characteristics

**Figure 1:**
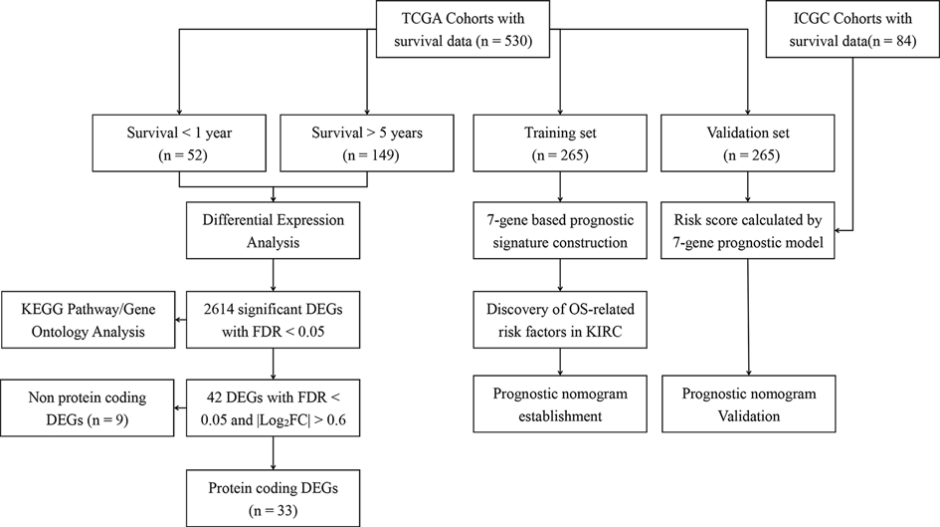
Flow chart of study design Abbreviation: KIRC, kidney renal clear cell carcinoma.

Figure 1 displayed the flow chart of this work. In all, 530 and 84 patients with full expression and clinical data were collected from TCGA and ICGC, respectively. The baseline characteristics of patients in the training set, validation set, and ICGC cohort are collected in Table 1.

**Table 1:** Clinical characteristics of ccRCC patients in the TCGA and ICGC datasets

Variables	TCGA cohort (*n*=530)		ICGC cohort (*n*=84) *N* (%)
	Training set (*n*=265) *N* (%)	Validation set (*n*=265) *N* (%)	
Status			
Alive	175 (66.04)	182 (68.68)	56 (66.67)
Dead	90 (33.96)	83 (31.32)	28 (33.33)
Age (years)	60.72 ± 12.84	60.40 ± 11.41	60.86 ± 9.68
Gender			
Male	166 (62.64)	178 (67.17)	45 (53.57)
Female	99 (37.36)	87 (32.83)	39 (46.43)
Stage			
I	133 (50.19)	132 (49.81)	48 (57.14)
II	28 (10.57)	29 (10.94)	12 (14.29)
III	61 (23.02)	62 (23.40)	15 (17.86)
IV	43 (16.22)	39 (14.72)	9 (10.71)
NA	0 (0.00)	3 (1.13)	0 (0.00)
Prior Malignancy			
Yes	37 (13.96)	35 (13.21)	NA
No	228 (86.04)	230 (86.79)	NA

Abbreviation: NA, not available.

### DEGs screening and functional enrichment analysis

**Figure 2:**
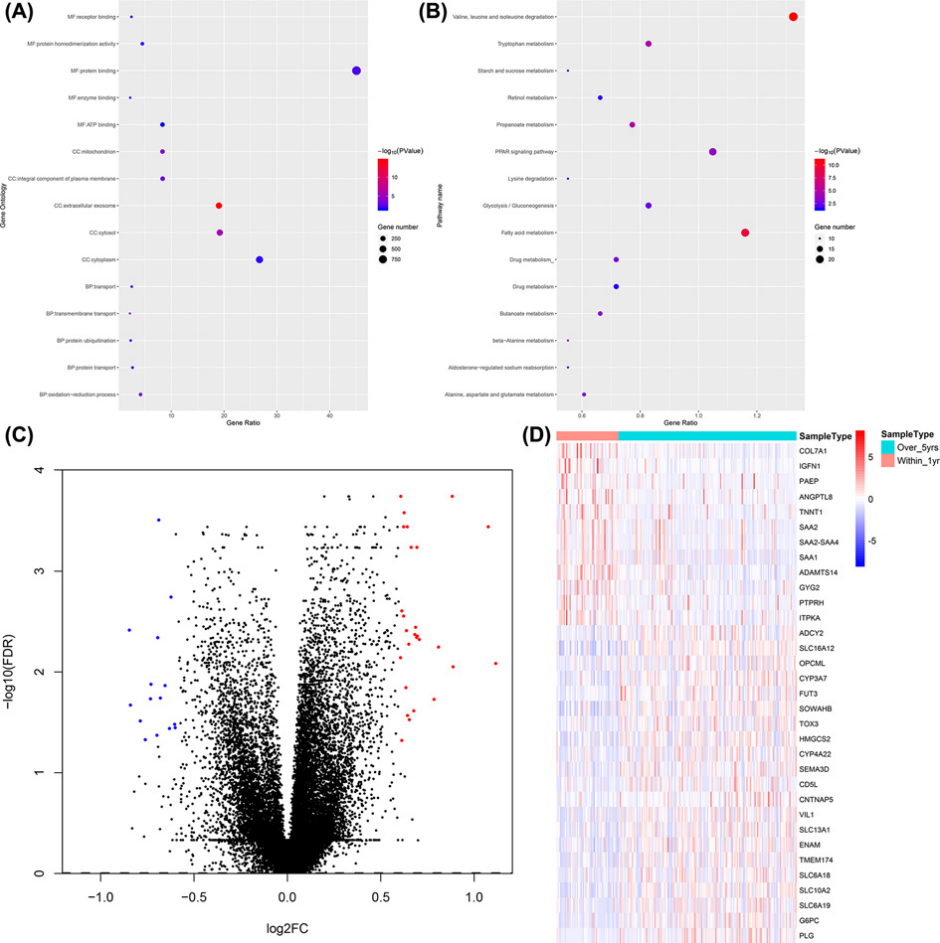
Identification and function enrichment analyses of the survival-related DEGs in the TCGA ccRCC cohort (**A**) Top 15 enriched GO terms of DEGs. (**B**) Top 15 enriched KEGG pathways of DEGs. (**C**) Volcano plot of DEGs: the abscissa represents |log2FC| and the ordinate represents −log10(FDR). The blue and red spots represent significantly down-regulated and up-regulated hub DEGs, respectively. (**D**) Cluster heatmap of the 33 hub DEGs.

By comparing 149 samples with OS > 5 years with 52 samples with OS < 1 year, 614 DEGs with criteria set as FDR < 0.05 were identified. GO and KEGG pathway enrichment analyses revealed the functions of these genes. The top 15 significantly enriched GO terms were gathered in Figure 2A and Supplementary Table S1, indicating that DEGs associated with pivotal terms such as the ‘oxidation-reduction process’ (GO category: biological process), ‘cytoplasm’ (GO category: cellular component), and ‘protein binding’ (GO category: molecular function). The top 15 significantly enriched pathways from the KEGG analysis were collected in Figure 2B and Supplementary Table S2, showing that DEGs participated mainly in pathways such as ‘valine, leucine and isoleucine degradation’, ‘fatty acid metabolism’, ‘PPAR signaling pathway’, ‘glycolysis/gluconeogenesis’, and ‘tryptophan metabolism’.

To narrow down the range, 42 hub DEGs with criteria set as FDR < 0.05 and |log_2_FC| > 0.60 were selected. As presented in Supplementary Table S3, ccRCC patients with longer OS were associated with 15 down- and 27 up-regulated hub DEGs. Furthermore, 9 non-coding genes were excluded, leaving 33 candidates for further analysis (volcano plot and heatmap were presented in Figure 2C,D, respectively).

### Seven-gene signature development

**Figure 3:**
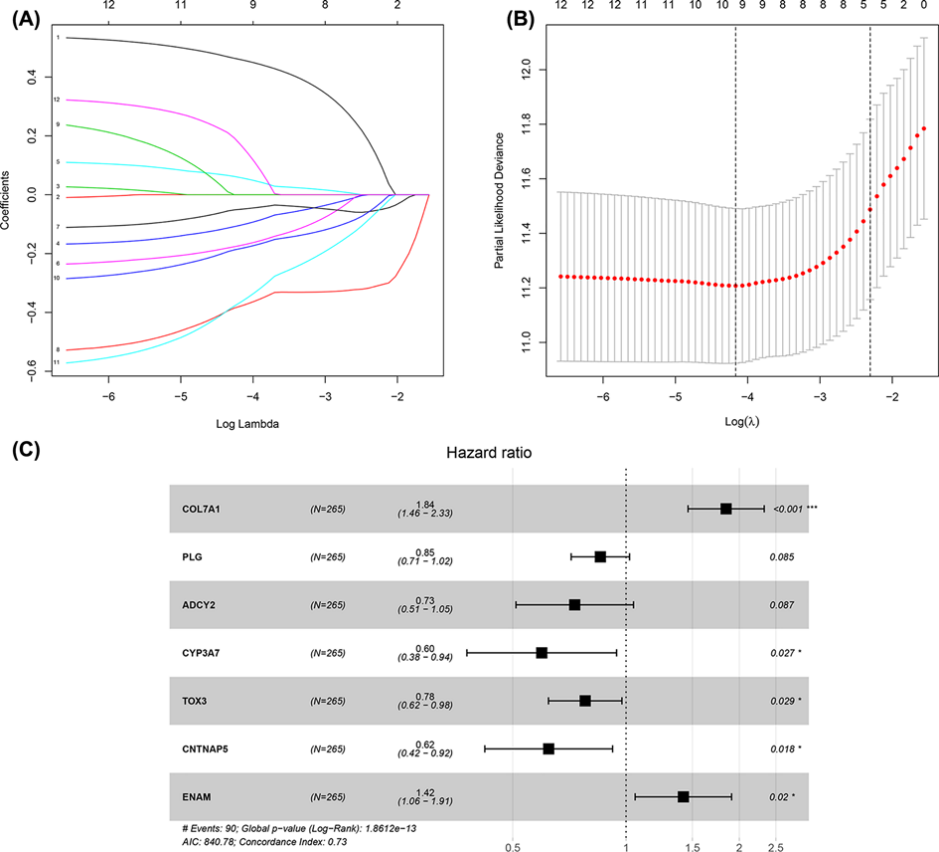
Risk score formula construction based on a seven-gene signature (**A**) The LASSO coefficient profiles of the 33 hub DEGs selected by Univariate Cox regression analysis. (**B**) Partial likelihood deviance for the LASSO coefficient profiles. (**C**) Forest plot based on Multivariate Cox regression results displays the HRs with corresponding 95% CIs of the seven genes selected by the LASSO model.

Based on univariate analysis, all 33 hub DEGs were significantly associated with ccRCC patients’ OS in the training set (*P*<0.05, Supplementary Table S4). Nine genes, including collagen type VII α 1 chain (COL7A1), plasminogen (PLG), inositol-trisphosphate 3-kinase A (ITPKA), adenylate cyclase 2 (ADCY2), solute carrier family 16 member 12 (SLC16A12), cytochrome P450 family 3 subfamily A member 7 (CYP3A7), TOX high mobility group box family member 3 (TOX3), contactin-associated protein family member 5 (CNTNAP5), and enamelin (ENAM), were identified as the most effective combination with the least components by LASSO-penalized Cox analysis (Figure 3A,B). Two genes (ITPKA and SLC16A12) were excluded based on the multivariate Cox regression model, and thus, a seven-gene prognostic signature was finally established (Figure 3C and Table 2). The risk score was calculated as follows:

$$\begin{array}{l} \text{Risk score }=\;-0.52\times \text{Exp(CYP3A7) }-0.47\times \text{Exp(CNTNAP5) }-0.31\times \text{Exp(ADCY2)}\\ \;-0.25\times \text{Exp(TOX3) }-0.16\times \text{Exp(PLG) }+0.35\times \text{Exp(ENAM) }+0.61\times \text{Exp(COL7A1)} \end{array}$$

**Table 2:** Outcomes of the multivariate Cox regression analysis of the seven genes identified by the LASSO-penalized model

Genes	Coefficient	HR (95% CI)	*P*-value
*CYP3A7*	−0.52	0.60 (0.38–0.94)	0.03
*CNTNAP5*	−0.47	0.62 (0.42–0.92)	0.02
*ADCY2*	−0.31	0.73 (0.51–1.05)	0.09
*TOX3*	−0.25	0.78 (0.62–0.98)	0.03
*PLG*	−0.16	0.85 (0.71–1.02)	0.08
*ENAM*	0.35	1.42 (1.06–1.91)	0.02
*COL7A1*	0.61	1.84 (1.46–2.33)	2.65E-07

Abbreviations: CI, confidence interval; HR, hazard ratio.

**Figure 4:**
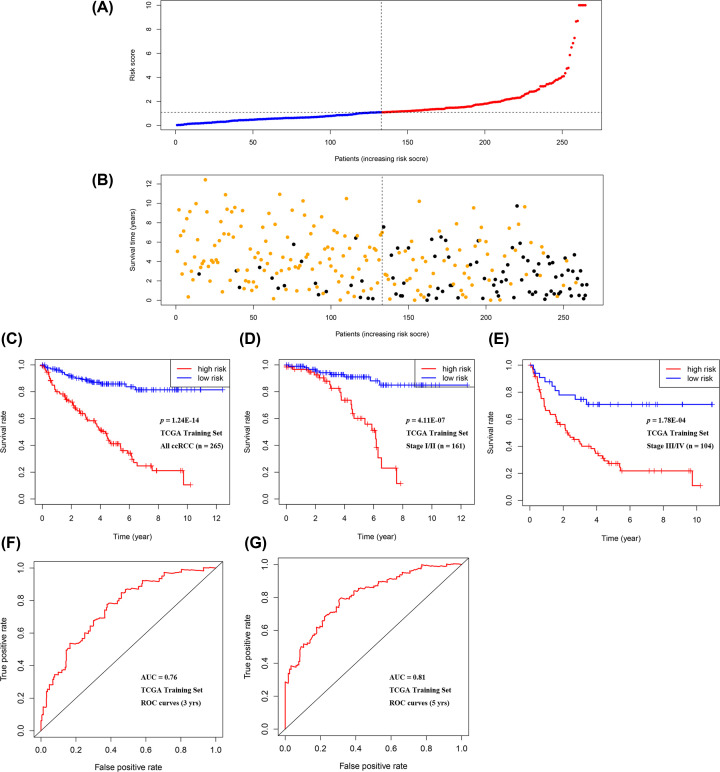
Preliminary evaluation of the predictive ability of the seven-gene signature in the training set (**A**) The seven-gene-based risk score distribution: using the median risk score as a cut-off point, patients were divided into a low-risk group (blue spots) and high-risk group (red spots). (**B**) The vital status of 265 patients: yellow and black spots represent alive and dead patients, respectively. (**C**–**E**) K–M survival curves of all patients’ OS (*n*=265), Stage I/II patients’ OS (*n*=161), Stage III/IV patients’ OS (*n*=104), respectively. (**F,G**) ROC curves for OS prediction based on the seven-gene signature within 3- and 5-years, respectively.

A higher risk score predicted worse survival. The distribution of risk scores and survival status of patients in the training set was exhibited in Figure 4A,B, respectively. Using the median score as the cut-off value, patients were classified into low-risk and high-risk groups. The Kaplan–Meier curves confirmed significantly better survival for low-risk groups than their high-risk counterparts (log-rank tests *P*<0.05, Figure 4C). Moreover, this advantage remained stable in both stage I/II and III/IV subgroups (Figure 4D,E). The ROC curves were plotted to assess the prognostic value of the seven-gene signature. The AUCs for 3- and 5-year OS predictions in the training set were 0.76 and 0.81 (Figure 4F,G). At the discovery stage, the preliminary result indicated the seven-gene signature achieved good performance in predicting OS using training set data.

### Construction and validation of the nomogram

**Figure 5:**
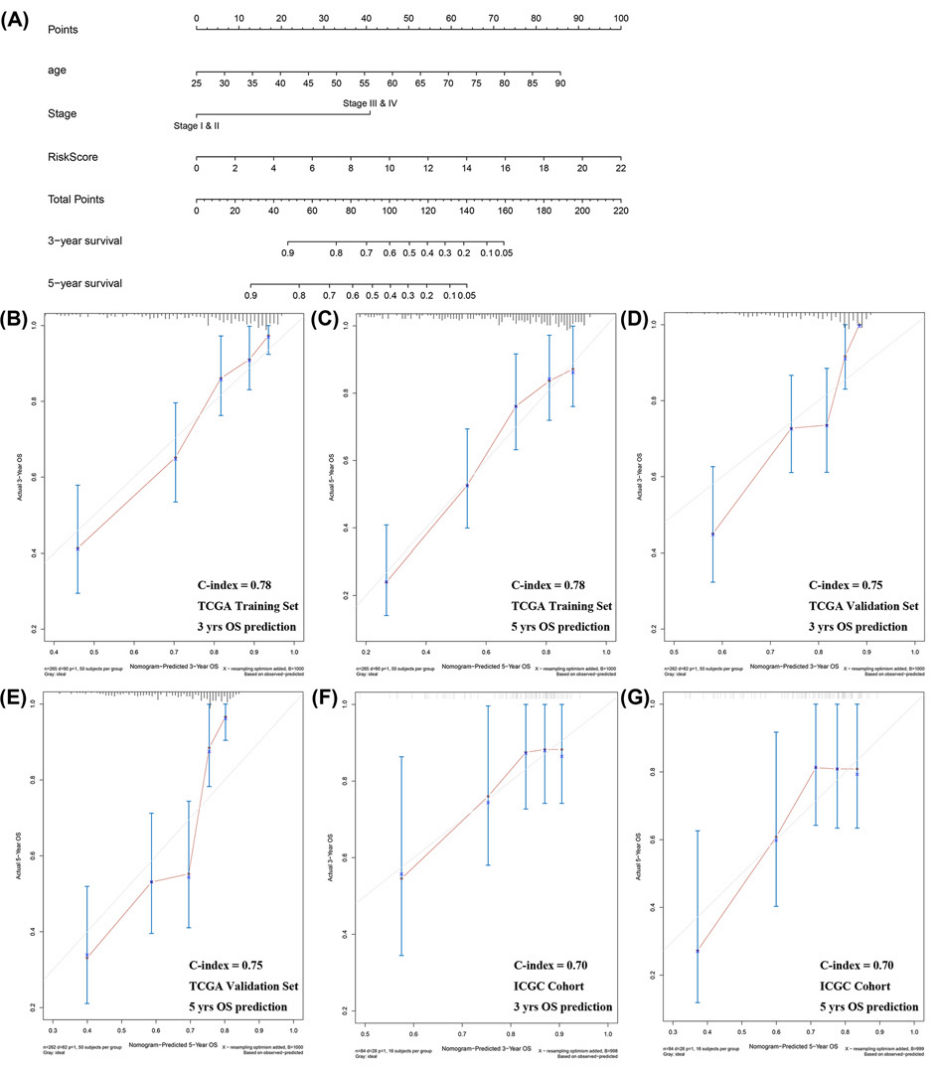
The establishment and assessment of a novel nomogram (**A**) A nomogram integrating clinical features with a seven-gene risk score for predicting of 3- and 5- years OS in patients with ccRCC. Calibration plots of the nomogram for 3- and 5- years OS prediction in the training set (**B,C**), internal validation set (**D,E**), and ICGC cohort (**F,G**), respectively. The abscissa represents the nomogram-predicted survival probability and the ordinate represents the actual survival.

In the training set, the univariate analysis indicated that age, TNM stage, and the seven-gene risk score impacted significantly on OS, whereas the gender and history of prior malignancy were found to be insignificant parameters (Table 3). The following multivariate analysis confirmed they were independent risk factors to OS of ccRCC patients (*P*-value for age, stage, and risk score were 1.91E-04, 1.49E-07, and 8.01E-08, respectively). The hazard ratios with 95% confidence intervals of age (elder versus young), stage (III/IV versus I/II), and risk score (high versus low) were 1.04 (1.02–1.06), 3.46 (2.18–5.49), and 1.15 (1.09–1.21), respectively. Subsequently, a nomogram predicting 3- and 5 years OS of ccRCC patients was constructed according to the multivariate analysis results of the training set (Figure 5A). The C-index for OS prediction of the nomogram was 0.78 (95% CI: 0.74–0.82). Internal validation using data from validation set revealed that a C-index of 0.75 (95% CI: 0.70–0.80). For external validation, C-index calculated using ICGC data was 0.70 (95% CI: 0.60–0.80). Besides, the calibration plots displaying the probability of 3- and 5 years survival indicated favorable curve-fitting between the nomogram-predicted outcomes and actual observation in the training set, validation set, and ICGC cohort, respectively (Figure 5B–G).

**Table 3:** Univariate and multivariate analyses of OS in the training set

Variables	Univariate analysis		Multivariate analysis	
	HR (95% CI)	*P*-value	HR (95% CI)	*P*-value
Age (years)	1.03 (1.02–1.05)	1.44E-04	1.04 (1.02–1.06)	1.91E-04
Gender				
Female	1			
Male	0.87 (0.57–1.33)	0.52	0.80 (0.52–1.24)	0.32
Stage				
I/II	1			
III/IV	3.72 (2.41–5.75)	3.46E-09	3.46 (2.18–5.49)	1.49E-07
Prior malignancy				
No	1			
Yes	0.85 (0.46–1.55)	0.59	1.01 (0.54–1.90)	0.98
Risk score	1.21 (1.15–1.26)	3.22E-15	1.15 (1.09–1.21)	8.01E-08

Abbreviations: CI, confidence interval; HR, hazard ratio.

### Comparison with previously reported prognostic tools

The predictive performance of our nomogram was compared with several reported prognostic tools, which were retrieved from PubMed database using ‘*(overall survival) AND (((c-index) AND signature) AND ((((clear cell renal cell carcinoma) OR renal clear cell carcinoma) OR clear cell carcinoma) OR KIRC))*’ as search terms. As presented in Table 4, clinical features such as TNM stage [12], Fuhrman grade [12], and the UISS risk model [24] alone seemed to be less competitive in terms of discrimination (c-indices were 0.65, 0.61, and 0.72, respectively). Similarly, using only gene signature such as the ClearCode34, a 34-gene signature model, was hardly satisfying when predicting OS in stage IV ccRCC patients [25]. The c-index of our nomogram was 0.78, second only to a 9-gene-signature-based nomogram reported by Wu et al. [26], which focused solely on stage III ccRCC (c-index: 0.79). Xiong et al. [24] reported a tool combining IGPI (immune-related 17 gene pairs index) with histologic grade and TNM stage for all ccRCC patients, with a c-index of 0.76. For localized ccRCC (stage I–III), Qu et al. [27] reported a tool combining four lncRNAs with the TNM stage, with a c-index of 0.73. In summary, by comprising only seven genes and two clinical features, our nomogram was economic and applicable to all stages of ccRCC without compromising the prognostic ability.

**Table 4:** Comparison of predicting performance with other reported prognostic tools

Study	Source	Stage	Size	Gene signature	Clinical feature in nomogram	C-index (95% CI)
Xiong et al., 2020	Hospital	All	101	IGPI consisting 17 gene pairs	Histologic grade, TNM stage	0.76
				-	UISS risk model only	0.72
Wu et al., 2019	TCGA	III	122	ATP6V1C2, PCSK1N, PREX1, ANK3, HLA-DRA, SELENBP1, TYRP1, GABRA2, SERPINA5	Age, ISUP grade, pN stage	0.79 (0.75–0.84)
Qu et al., 2018	TCGA	I–III	444	ENSG00000255774, ENSG00000248323, ENSG00000260911, ENSG00000231666	TNM stage	0.73 (0.65–0.81)
Develasco et al., 2017	TCGA	IV	54	ClearCode34	-	0.63 (0.51–0.75)
Tang et al., 2019	Hospital	All	140	-	TNM stage only	0.65 (0.56–0.74)
				-	Fuhrman grade only	0.61 (0.52–0.70)
The present study	TCGA	All	530	CYP3A7, CNTNAP5, ADCY2, TOX3, PLG, ENAM, COL7A1	Age, TNM stage	0.78 (0.74–0.82)

Abbreviations: ClearCode34, a 34-gene signature model; IGPI, immune-related gene pair index.

## Discussion

In this work, we developed an OS-related seven-gene signature in ccRCC, namely CYP3A7, CNTNAP5, ADCY2, TOX3, PLG, ENAM, and COL7A1, from TCGA training set. The ensuing univariate and multivariate Cox regression indicated that the patient’s age, TNM stage, and the seven-gene risk score were independent prognostic factors to OS and a nomogram was then constructed. Subsequently, C-indices and the curve-fitting calibration plots of the training set, internal validation set, and ICGC ccRCC cohort demonstrated the decent predictive performance of the nomogram.

To the best of our knowledge, this is the first study that revealed the putative protective role of CYP3A7 in the prognosis of ccRCC. Members of the cytochrome P450 superfamily are a group of metalloproteins that involve in metabolic biotransformation of endogenous and exogenous substrates, including carcinogens [28]. Conversely, overexpression of CYP3A7 was witnessed in hepatocellular carcinoma [29,30], suggesting that it might exert varied functions among different types or stages of tumor. Belonging to the neurexin family, the product of CNTNAP5 functioned as cell adhesion molecules in the nervous system and participated in diseases such as autism, Alzheimer’s disease, and schizophrenia [31–33]. The expression level of CNTNAP5 in the kidney is relatively low but still detectable, and only one study reported a SH3KBP1–CNTNAP5 fusion in upper tract urothelial carcinoma [34]. A higher level of ADCY2 was shown to connect to longer OS in our study, but few studies reported on its role in tumor progression. Reportedly, ADCY suppressed migration and invasion of pancreatic tumor cells by increasing the level of second messenger cyclic adenosine monophosphate (cAMP), however, ADCY2 was found to be down-regulated in pancreatic tumor tissues [35]. TOX3 has been newly identified as a ccRCC suppressor gene as it inhibited tumor cell migration and invasion by repressing the SNAIL members SNAI1 and SNAI2 at the transcriptional level [36]. This was consistent with our results that TOX3 exerted protective influence in ccRCC. A lower level of PLG in ccRCC patients with shorter survival, higher stages and grades were described in several studies [37–39], consistent with our finding that PLG served as a protective factor. Our outcomes illustrated that patients with OS > 5 years had significantly up-regulated ENAM when compared with those with OS < 1 year. Similarly, Bhalla et al. [40] reported a lower expression of ENAM in late-stage ccRCC when compared with those in the early stage. When extending to patients with all lengths of OS in the training set, however, multivariate Cox regression yielded the opposite conclusion that ENAM was a risk factor to the OS of ccRCC. Thus, ENAM may have a more complex role in the progression of ccRCC. Coding for type IV collagen, COL7A1 was also a risk factor to ccRCC survival according to our results, supported by a previous study revealing high expression of COL7A1 was associated with tumor invasion and shorter survival in several types of squamous cell cancer [41–43].

Prevalence of targeted and individualized therapy calls for novel prognostic tools integrating genetic signatures with clinical features to improve risk assessment and stratification. The comparison of c-indices revealed that the present nomogram based on an OS-related seven-gene risk score, age, and TNM stage has better predictive accuracy than the traditional prognostic tools such as the TNM staging system, Fuhrman grading system, UISS risk model, ClearCode34. Furthermore, the present nomogram had undergone both internal and external validation and showed good reproducibility. In terms of clinical significance, the present work suggested that patients with higher points calculated according to our nomogram might benefit from more active surveillance as well as adjuvant treatments such as tyrosine kinase inhibitors [44] and immunotherapy [45].

Limitations of the present work should be notified. The first is the retrospective design of the present study. Second, the seven genes were less reported in ccRCC. Third, the external validation cohort in the present study comprising merely 84 samples. Hence, further experiments are warranted to elucidate the roles of the seven genes in ccRCC development and progression and to validate the predictive ability of the nomogram in prospective studies with a larger population.

## Conclusions

In the present study, we excavated seven novel OS-related genes (CYP3A7, CNTNAP5, ADCY2, TOX3, PLG, ENAM, and COL7A1) from TCGA and used them to build a formula for risk score calculation. Besides, by integrating the seven-gene signature and clinical features (age and TNM stage), we proposed and validated a nomogram for OS prediction in ccRCC which might have promising application prospects.

## Supplementary Material

Supplementary Tables S1-S4

## Abbreviations

ADCY2: adenylate cyclase 2
AUC: area under the curve
ccRCC: clear cell renal cell carcinoma
CNTNAP5: contactin-associated protein family member 5
COL7A1: collagen type VII α 1 chain
CYP3A7: cytochrome P450 family 3 subfamily A member 7
C-index: Harrell’s concordance index
DEG: differentially expressed gene
ENAM: enamelin
FC: fold change
FDR: false discovery rate
GO: Gene Oncology
ICGC: International Cancer Genome Consortium
ITPKA: inositol-trisphosphate 3-kinase A
KEGG: Kyoto Encyclopedia of Genes and Genomes
LASSO: least absolute shrinkage and selection operator
OS: overall survival
PLG: plasminogen
RCC: renal cell carcinoma
ROC: receiver operating characteristic
SLC16A12: solute carrier family 16 member 12
TCGA: The Cancer Genome Atlas
TNM: Tumor, lymph Node, and Metastasis staging system
TOX3: TOX high mobility group box family member 3
UISS: University of California Integrated Staging System
