# Supplementary material for: Profiles of overall survival-related gene expression-based risk signature and their prognostic implications in clear cell renal cell carcinoma
Source: Biosci Rep. 2020 Sep 16;40(9):BSR20200492. doi: 10.1042/BSR20200492 (PMC7494988; doi:10.1042/BSR20200492)
Supplement: Supplementary Tables S1-S4 [file BSR-2020-0492_supp.pdf]

Category	Term	Count	*p*-value
BP	oxidation-reduction process	88	2.78E-04
BP	protein transport	56	0.01
BP	transport	53	3.08E-03
BP	protein ubiquitination	49	0.03
BP	transmembrane transport	46	5.93E-05
CC	cytoplasm	561	0.01
CC	cytosol	403	3.28E-06
CC	extracellular exosome	400	2.91E-15
CC	integral component of plasma membrane	176	1.18E-03
CC	mitochondrion	175	7.89E-05
MF	protein binding	947	5.11E-03
MF	ATP binding	175	0.03

MF	protein homodimerization activity	95	0.01
MF	receptor binding	52	6.65E-03
MF	enzyme binding	47	0.02

Table S1. Top 15 enriched GO terms of the DEGs.

GO: gene ontology; DEG: differentially expressed gene; BP: biological process; CC: cellular component; MF: molecular function.

Table S2. Top 15 enriched KEGG pathways of the DEGs.

Pathway ID	Name	Count	*p*-value	Genes
hsa00280	Valine, leucine and isoleucine degradation	24	1.03E-11	BCKDHA, ACAA2, ALDH6A1, ACADSB, ACADM, EHHADH, BCKDHB, ACAT1, HIBADH, ALDH3A2, HADHA, HADHB, AUH, MCCC2, DBT, ALDH7A1, MUT, HMGCS2, AOX1, ALDH2, ABAT, HIBCH, HADH, PCCA
hsa00071	Fatty acid metabolism	21	6.78E-10	ACAA2, ACOX1, ACADSB, ACADM, CPT2, CHKB, EHHADH, ADH5, ADH6, ADH5P4, ACADL, ACAT1, ALDH3A2, HADHA, CPT1A, HADHB, CYP4A11, ALDH7A1, CYP4A22, ACSL1, ALDH2, HADH
hsa03320	PPAR signaling pathway	19	2.96E-04	ACOX1, PPARA, ACADM, CPT2, CHKB, EHHADH, PPARG, ACADL, CPT1A, PCK1, CYP4A11, ACSL1, CYP4A22, HMGCS2, FABP1, SCD5, SLC27A2, SCP2, PLTP
hsa00010	Glycolysis / Gluconeogenesis	15	4.22E-03	ALDOB, ADH5, FBP1, ADH6, BPGM, DLAT, ADH5P4, ALDH3A2, PCK1, ALDH7A1, GALM, G6PC, HK3, PKLR, ALDH2, ENO3
hsa00380	Tryptophan metabolism	15	4.43E-05	DDC, EHHADH, OGDHL, ACMSD, WARS2, OGDH, ACAT1, ALDH3A2, HADHA, ALDH7A1, AOX1, HAAO, ALDH2, CAT, HADH
hsa00640	Propanoate metabolism	14	1.30E-05	ALDH6A1, ACADM, EHHADH, ACSS3, ACAT1, ALDH3A2, HADHA,

				ALDH7A1, MUT, ALDH2, ABAT, HIBCH, SUCLA2, PCCA
hsa00982	Drug metabolism	13	0.03	FMO4, CYP3A4, GSTA1, UGT1A9, CYP3A7, FMO1, FMO2, AOX1, ADH5, ADH6, UGT2A3, ADH5P4, UGT2B7, MGST2
hsa00983	Drug metabolism	13	1.59E-03	CYP3A4, CES2, CYP3A7, NAT1, UPB1, UPP1, TPMT, UGT1A9, ITPA, UCK2, UGT2A3, IMPDH1, UGT2B7
hsa00830	Retinol metabolism	12	0.03	CYP3A4, RDH12, CYP4A11, UGT1A9, CYP3A7, CYP4A22, ADH5, ADH6, UGT2A3, PNPLA4, ADH5P4, UGT2B7, RETSAT
hsa00650	Butanoate metabolism	12	6.37E-04	ACSM3, ALDH7A1, HMGCS2, ALDH5A1, EHHADH, ALDH2, ABAT, BDH2, HADH, ACAT1, ALDH3A2, HADHA
hsa00250	Alanine, aspartate and glutamate metabolism	11	1.15E-03	GOT1, GLUD2, ACY3, ALDH5A1, GLUD1, GFPT2, ABAT, AGXT2, ASNS, AGXT, DDO
hsa00310	Lysine degradation	10	0.04	ALDH7A1, EHHADH, OGDHL, ALDH2, OGDH, HADH, ACAT1, ALDH3A2, HADHA, BBOX1
hsa00500	Starch and sucrose metabolism	10	0.03	GBA3, G6PC, UGT1A9, ENPP3, HK3, MGAM, TREH, UGT2A3, AGL, UGT2B7
hsa04960	Aldosterone-regulated sodium reabsorption	10	0.03	ATP1B1, SGK1, PIK3CB, NR3C2, ATP1A1, NEDD4L, PIK3R3, INSR, SLC9A3R2, PIK3R1
hsa00410	beta-Alanine metabolism	10	2.73E-04	ALDH7A1, ACADM, SRM, UPB1, EHHADH, ALDH2, ABAT, HIBCH,

	ALDH3A2, HADHA

KEGG: Kyoto encyclopedia of genes and genomes; DEG: differentially expressed gene.

Table S3. The 42 hub DEGs.

Gene symbol	Log_2FC	FDR	Regulation
COL7A1	-0.85	0.00	Down
IGFN1	-0.84	0.02	Down
PAEP	-0.79	0.03	Down
ANGPTL8	-0.76	0.05	Down
TNNT1	-0.73	0.02	Down
SAA2	-0.73	0.01	Down
SAA2-SAA4	-0.70	0.04	Down
SAA1	-0.69	0.00	Down
ADAMTS14	-0.69	0.00	Down
GYG2	-0.68	0.02	Down
†AL357992.1	-0.65	0.01	Down
PTPRH	-0.63	0.04	Down
ITPKA	-0.62	0.00	Down
†AC116614.1	-0.60	0.03	Down

†LINC01914	-0.60	0.04	Down
ADCY2	0.61	0.01	Up
SLC16A12	0.61	0.00	Up
OPCML	0.61	0.05	Up
†AP000439.2	0.61	0.00	Up
CYP3A7	0.62	0.00	Up
FUT3	0.62	0.00	Up
SOWAHB	0.63	0.00	Up
TOX3	0.64	0.01	Up
HMGCS2	0.64	0.00	Up
†AC026462.3	0.64	0.00	Up
CYP4A22	0.64	0.03	Up
SEMA3D	0.65	0.01	Up
CD5L	0.65	0.03	Up
†AC124854.1	0.66	0.00	Up
CNTNAP5	0.68	0.02	Up

†ENPP7P8	0.68	0.00	Up
VIL1	0.69	0.00	Up
SLC13A1	0.69	0.00	Up
ENAM	0.69	0.00	Up
TMEM174	0.70	0.00	Up
†LINC00113	0.71	0.00	Up
SLC6A18	0.79	0.02	Up
†RNA5SP107	0.81	0.01	Up
SLC10A2	0.88	0.00	Up
SLC6A19	0.89	0.01	Up
G6PC	1.08	0.00	Up
PLG	1.12	0.01	Up

DEG: differentially expressed gene; FC: Fold change; FDR: False discovery rate. Bold fonts indicated genes with protein-coding function. † Genes without protein-coding function.

Table S4. Results of univariate Cox regression analysis of the 33 hub DEGs.

Id	HR	HR.95L	HR.95H	*p* value
COL7A1	1.76	1.39	2.24	0.00
IGFN1	1.34	1.09	1.64	0.01
PAEP	1.15	1.03	1.28	0.01
ANGPTL8	1.16	1.03	1.30	0.02
TNNT1	1.19	1.03	1.37	0.02
SAA2	1.10	1.01	1.21	0.03
SLC6A18	0.88	0.79	0.99	0.03
SLC10A2	0.80	0.71	0.90	0.00
SLC6A19	0.84	0.76	0.93	0.00
G6PC2	0.46	0.26	0.81	0.01
PLG	0.72	0.61	0.85	0.00
SAA1	1.09	1.03	1.16	0.00

ADAMTS14	1.43	1.13	1.81	0.00
GYG2	1.32	1.05	1.65	0.02
PTPRH	1.21	1.03	1.42	0.02
ITPKA	1.39	1.18	1.64	0.00
ADCY2	0.53	0.38	0.74	0.00
SLC16A12	0.77	0.70	0.85	0.00
OPCML	0.77	0.64	0.92	0.00
CYP3A7	0.38	0.27	0.53	0.00
FUT3	0.68	0.52	0.89	0.00
SOWAHB	0.64	0.54	0.75	0.00
TOX3	0.65	0.53	0.79	0.00
HMGCS2	0.87	0.80	0.96	0.00
CYP4A22	0.86	0.75	0.98	0.03
SEMA3D	0.77	0.63	0.93	0.01

CD5L	0.80	0.67	0.96	0.02
CNTNAP5	0.45	0.32	0.65	0.00
VIL1	0.87	0.77	0.99	0.03
SLC13A1	0.88	0.79	0.97	0.01
ENAM	0.64	0.52	0.79	0.00
TMEM174	0.88	0.80	0.97	0.01
SAA2-SAA4	1.12	1.01	1.23	0.03
